# Experiences and Perceptions of Self‐Harm in Rural‐Dwelling Adults: A Rapid Review of Qualitative Evidence

**DOI:** 10.1111/hex.70268

**Published:** 2025-04-25

**Authors:** Katie Saunders, William Nicholls, Nadia Corp, Tom Kingstone, Faraz Mughal, Carolyn A. Chew‐Graham, Jane Southam, Tamsin Fisher

**Affiliations:** ^1^ School of Medicine Keele University Staffordshire UK; ^2^ Midlands Partnership University NHS Foundation Trust Staffordshire UK

**Keywords:** help‐seeking, literature review, qualitative methods, rurality, self‐harm, suicide

## Abstract

**Background:**

Self‐harm is associated with factors that are relevant to and exacerbated by rurality. Living in rural areas may intensify existing socio‐economic disadvantages linked to service access, employment opportunities, transport conditions and risks from hazardous environments. Geographical isolation and fragmented social networks, particularly those related to family, are also common among rural residents. Rurality is therefore likely to shape experiences of mental health problems, including self‐harm. However, this literature has not been synthesised.

**Aim:**

To synthesise current qualitative evidence on the experiences and perceptions of self‐harm among rural‐dwelling adults and care providers' perspectives and to identify knowledge gaps.

**Approach:**

Rapid review of qualitative evidence identified via relevant electronic literature databases. Thematic synthesis was used to compare findings on perceptions and experiences of self‐harm in rural areas. Confidence in synthesis findings was assessed using GRADE‐CERQual.

**Findings:**

Searches identified 1673 unique references, of which 14 were included in the final synthesis. Two themes were generated with high to moderate confidence: ‘experiences of rural self‐harm’ with two subthemes ‘reasons for self‐harm’ and ‘perceptions of self‐harm’, and ‘access to healthcare’ with two subthemes: ‘healthcare practitioners' perceptions of rural self‐harm’ and ‘lack of support and resources’. Various reasons and motivations for self‐harm were identified; stigma was commonly reported. Services for rural residents who self‐harmed were difficult to access. Healthcare practitioners in these areas may lack adequate training, which may maintain stigmatised views.

**Conclusion:**

The review identified shared experiences and motivations for self‐harm across different rural contexts globally. Perceptions of self‐harm by people with lived experience, family and healthcare professionals reflected stigmatised views, which impacted access to and provision of care. Experiences and perceptions of self‐harm reported in the literature are somewhat overshadowed by data on suicide and suicide behaviours. Methodological implications are noted in terms of the complexity of extracting data about self‐harm. Future research would help inform intervention development for people who are at risk of self‐harm, to support healthcare practitioners to improve awareness and identify best practices to support those who self‐harm.

**Patient or Public Contribution:**

Patient and public involvement was integrated at various points throughout the study, including reviewing themes, supporting the writing up of findings and a review draft and final manuscripts.

## 1 Background

Self‐harm is defined as an intentional act of self‐poisoning or self‐injury, often as an expression of emotional distress [1, 2]; this definition is used throughout this review. A history of self‐harm is reported in many people who die by suicide but, whilst both are global health concerns and share similar risk factors, it is important to treat these as distinct concepts [3, 4]. Global estimates suggest that 14.6 million people are affected by self‐harm each year, although the incidence of self‐harm is likely to be under‐reported [3]. Self‐harm is more common among people 15–35 years of age [5]; gender differences are described, with rates of self‐harm peaking at 16–24 years of age for women and 25–34 years of age among men [3, 4]. In addition to age and gender, several other risk factors are associated with self‐harm, such as mental health disorders like depression, physical health conditions, socio‐economic disadvantage, social isolation, ethnicity and experiencing stressful life events [3, 6]—the interplay between these factors is likely to be important. Geographical location may heighten the risk of depression linked to social isolation and loneliness; these are important predictors for self‐harm behaviours [7, 8, 9].

Forty‐three percent of the global population lives in rural areas [10]. Rurality and mental health share a complex relationship [1]. Where health and well‐being are concerned, favourable comparisons are often drawn between rural residents and their urban counterparts, with rural residents reported to live ‘healthier’ lives, to feel part of cohesive communities, to benefit from increased access to green space and to have a greater sense of belonging—all of which are protective factors for mental health [11, 12]. The reality for some, however, may be very different. Rural residents experience disparities in access to healthcare services, commonly referred to as an ‘accessibility deficit’, and is due to coalescing factors including a lack of physical presence of health services in rural areas, poor public transport and limited mental health training in healthcare staff in rural areas [13, 14, 15, 16, 17]. Mental health problems experienced by people living in rural areas are under‐researched, perhaps protected by positive notions of rurality and presumed access to green spaces [18].

Suicide in rural areas has received more attention in research than self‐harm. The global incidence of suicide is reported to be relatively high in rural areas [19] including Taiwan, Ireland, Australia, Scotland, Canada, New Zealand and Finland [20]. Data from the United States report that suicide in rural communities to be 1.5 times higher than in urban communities [21]. Data for rural self‐harm are lacking but are likely to follow a similar pattern given the relationship and shared risk factors. A recent literature review identified interpersonal support, social victimisation and under‐reporting of mental health disorders as key social factors contributing to high rates of rural suicide [22]. Higher suicide rates have been consistently reported among farmers [23, 24, 25]. A recent synthesis of qualitative research on suicide among farmers described themes of identity, finances, familial relationships, isolation and access to lethal means (e.g., agricultural pesticides and firearms) as contributing factors—the paper does not consider non‐suicidal self‐harm or the wider rural population [23]. Rural residents may have greater exposure to suicidality and suicide, due to higher incidence rates in farming and access to lethal means [16, 17, 26, 27]. Financial difficulties, which may be attributed to rural poverty and the relative lack of employment opportunities in rural areas, are also associated with suicidality [22]. A synthesis of qualitative evidence on self‐harm in rural areas has not been conducted.

The main aim of this rapid review was to synthesise qualitative evidence, including lived experience and care provider perspectives, self‐harm among adults living in rural areas and to identify social and environmental factors that influence access to care. This review will consolidate existing qualitative evidence and conceptual understanding to better define the distinct features (perceptions and experiences) of rural self‐harm. A secondary aim was to identify gaps in knowledge and opportunities for future interventions for rural adults at risk of self‐harm.

## 2 Methods

This rapid review was informed by the Cochrane guidance for rapid reviews [28]. The rapid review was deemed an appropriate choice as it provides a means to synthesise data effectively in a shorter timeframe [29]. The review was reported according to the Preferred Reporting Items for Systematic Reviews and Meta‐Analyses (PRISMA) guidance [30] and Enhancing Transparency in Reporting the Synthesis of Qualitative Research (ENTREQ) [31] checklists. The protocol was registered with the International Prospective Register of Systematic Reviews (PROSPERO; CRD42023439855).

### 2.1 Search Strategy

Electronic database searches were conducted by N.C. using MEDLINE, EMBASE, PsycINFO, CINAHLPlus, ASSIA and Web of Science (Science Citation Index, Social Science Citation Index). Searches were run from inception to 26 June 2023. See Supporting Information S1 for database searches. The results of each search were downloaded into Endnote 20 for deduplication. A final set of unique search results was then imported into Rayyan for screening.

### 2.2 Eligibility Criteria

The inclusion and exclusion criteria were used to guide the selection of relevant papers, see Supporting Information S2: Table 1. Self‐harm is an umbrella term for a form of self‐injurious behaviour regardless of the intent and includes both non‐suicidal self‐injury (NSSI) and deliberate self‐harm (DSH) [2]. Although NSSI is self‐injury without suicidal intent, NSSI is a significant predictor for suicide and attempted suicide [4], with those that engage in NSSI being more likely to have suicidal ideation and attempt suicide than those that do not [32]. However, attempted suicide and suicide are not predictors for self‐harm [33]. This framework directed our literature search; research that discussed suicide without mentioning a history of self‐harm was not included in our search, but those that mentioned a history of self‐harm were included. Further, there is a conceptual overlap with suicide; self‐harm may or may not be carried out with suicidal intent, but all forms of suicide may be considered acts of self‐harm. Despite conceptual overlap, we were mindful not to conflate these two phenomena.

### 2.3 Study Selection

One author (W.N.) completed title and abstract screening against the predefined eligibility criteria and excluded those not relevant. Full texts were retrieved for the remaining papers, 10% of these were screened for inclusion by T.F. and all papers were reviewed by W.N. All excluded papers were checked and confirmed by a second reviewer (T.F.). Any disagreements on study eligibility were resolved through discussion with a third reviewer (T.K.).

### 2.4 Data Extraction and Quality Appraisal

A bespoke data extraction form was created in Microsoft Excel, piloted and used to record data for analysis. The form included columns for the following: author, year, country, study design, aim, population, setting sample, proportion of rural, methods and findings. One author (W.N.) completed the initial data extraction and another author (K.S.) checked this before synthesis commencing.

A quality assessment was performed, alongside data extraction, using the Mixed Methods Appraisal Tool (MMAT) [34]. Results are scored out of 5 for a qualitative study and out of 10 for a mixed‐methods study; those that scored below 2/5 or 4/10 (40%) were considered weak studies and were excluded after confirmation by T.K. and T.F. Two authors (W.N., K.S.) independently completed the quality appraisal process. A third author (T.F.) resolved any differences through discussion. Due to the focus on experiences and perceptions, the review design sought to identify mixed‐methods studies that included a qualitative component; quantitative data noted in mixed‐methods studies were not included in the findings.

### 2.5 Thematic Synthesis

As the aim of this rapid review was to understand experiences and identify relevant social and environmental factors that influence self‐harm behaviours and healthcare interactions, a thematic synthesis was conducted [35]. Like other methods to analyse primary and secondary data, thematic synthesis includes the generation of higher order codes and themes using a systematic approach [36, 37]. Data from findings sections of included papers were used in a three‐stage process which was completed by one author (K.S.): line‐by‐line coding—where each line of data was inductively coded; descriptive themes were developed—similar codes were compared and grouped; analytical themes were then developed—these were formed from an enhanced level of interpretation and abstraction, compared to the descriptive codes [35, 36].

Descriptive themes and corresponding data extracts were reviewed in multidisciplinary research team meetings to agree with analytical themes and support refinement through discussion. Team meetings brought mixed perspectives together to generate rich discussion; perspectives spanned psychology (K.S.), sociology (T.K.), social geography (T.F.), clinical education and practice (C.A.C.‐G., F.M. and W.N.), information science (N.C.) and lived experience of mental health problems (J.S.).

### 2.6 Confidence in Synthesis

The Grades of Recommendation, Assessment, Development, and Evaluation Confidence in the Evidence from Qualitative Reviews (GRADE‐CERQual) approach was used to ascertain confidence in the synthesised findings [38].

### 2.7 Patient and Public Involvement

The design of this study and the development of the research question were informed through patient and public involvement (PPI). A group of four people with either lived experience of mental health problems (depression, self‐harm and suicide) or as a family caregiver. The group helped refine the original research question, provided feedback on preliminary findings from the synthesis and identified key messages for public audiences. The group acknowledged the need for suicide and self‐harm to be distinguished in the findings, where possible, although they recognised the difficulty of doing this in the included data. One member of the group (J.S.) supported the drafting of this paper.

## 3 Results

### 3.1 Study Flow

**Figure 1:**
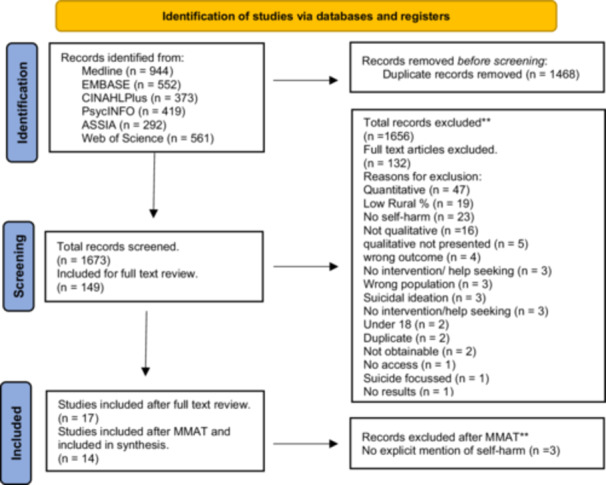
PRISMA flow chart of studies sources, screened and used in review.

The database search retrieved 1673 unique papers, of which 149 were included for full‐text screening. Of these, 17 met the criteria and were included for data extraction; 132 papers were excluded. One paper was excluded based on quality appraisal using the MMAT [34] due to its score of 2/5 (40%), three were then excluded during analysis as there was no explicit mention of self‐harm. Thus, 14 studies were included in the synthesis. See Figure 1 for the PRISMA study flow diagram.

### 3.2 Characteristics of Included Studies

Selected studies were published between 1999 and 2023 and were conducted in the United States (*n* = 3), Canada (*n* = 1), China (*n* = 1), Ethiopia (*n* = 1), Australia (*n* = 5), South Africa (*n* = 1) and Sri Lanka (*n* = 2), with 1580 participants in total across the 14 studies. There were 2 mixed‐methods and 14 qualitative studies that used focus groups (*n* = 4), semi‐structured interviews (*n* = 5), structured interviews (*n* = 3), case studies (*n* = 1) and photovoice methods (*n* = 1). Sample sizes ranged from 3 to 792, with a mean of 84.5 (SD 181.54) across the studies. In total, 13 studies described the population as rural (*n* = 13) or ‘small town’ (*n* = 1), and one as a mix of both rural and urban populations. Full characteristics of included studies are described in Supporting Information S2: Table 3.

During data extraction and screening, overlap in the reporting of self‐harm and suicide in the published research was noted. In some instances, data on self‐harm were often mixed with suicide, which made extraction challenging. Thus, we adopted a broader view of self‐harm to include historical experiences of self‐harm before a suicide, NSSI and self‐poisoning.

### 3.3 Quality Appraisal

Using the MMAT [34], most studies were of a high quality with strong scores at 5/5 for qualitative studies (*n* = 12) and 10/10 for one mixed methods (*n* = 1), with one study being moderate‐weak at 5/10 for mixed methods, and one study was moderate‐strong as a qualitative study and one study scores 2/5 which was considered weak.^1^ Full MMAT results can be seen in Supporting Information S2: Table 2.

### 3.4 Final Analytical Themes and Confidence in Synthesis (GRADE‐CERQual)

Two themes were generated: (1) Experiences of rural self‐harm, which has two subthemes: reasons for self‐harm and perceptions of self‐harm; (2) access to healthcare for rural self‐harm, with two subthemes: healthcare provider perceptions of self‐harm and a lack of support and resources. Themes were generated at a descriptive level, to reflect the depth of the reported data, and selected to connect findings across the evidence building upon themes reported in the included papers. Details of the theme development process can be found in Supporting Information S2: Table 4. Themes are presented with data extracts to evidence our interpretations.

GRADE‐CERQual ratings of confidence are described in full in Supporting Information S2: Table 5; each theme demonstrated moderate to high confidence. For each theme, the contributing articles are numbered against the theme heading (see Supporting Information S2: Table 3 for article numbers).

### 3.5 Experiences of Rural Self‐Harm

This theme not only describes experiences of self‐harm in rural communities and possible reasons and motivations for engaging in self‐harm but also highlights the perceptions of self‐harm by those that reside and work within these rural communities. The theme explores experiences in relation to suicidality and mental health and ill‐health.

#### 3.5.1 Reasons for Self‐Harm (High Confidence) [1–12 and 14]

The included papers suggest that people within rural communities may engage in self‐harm for a multitude of reasons. Mental illness, family and or financial problems were posited for why some individuals who attended the hospital:

Sometimes the reason for self‐harm is a mental illness or it can be a family or financial problem.

(Senarathna et al. [16, p. 4])

A decline in community support leading to loneliness and isolation was not only perceived as motivation for self‐harm but also a significant predictor for poor mental health outcomes in rural communities:

Participants described deteriorating sense of community, as well as increasing loneliness and alienation associated with poor mental health and self‐harm or suicide.

(George et al. [15, p. 6])

Participants described decades‐long reductions in organisation presence and neighbourly interaction… an isolating dynamic that produced growing distrust…

(George et al. [15, p. 7])

Occupation type was identified as contributing to the sense of isolation. The following extract identifies isolation in the context of farming and suicide risk; this could, however, apply as much to self‐harm:

The isolating nature of farming itself and the many hours of working alone were also discussed. Participants in a number of groups spoke about the combination of geographical and emotional isolation as a potential risk factor for suicide.

(Perceval et al. [13, p. 281])

Illness experiences in the context of long‐term conditions such as chronic pain and diabetes could impact work and family, which in turn may contribute to self‐harm behaviours:

Four of the five survivors [of attempted suicide] reported being on medication for conditions such as tuberculosis as well as lifestyle‐related diseases such as diabetes, asthma and high blood pressure… these factors add burden on family relations as it has financial implications.

(Holtman et al. [39, 303] [text added not in original])

Some participants reflected on difficult marital situations before an individual taking their own life, and how it was believed this may have been a contributing factor:

She believed that marital distress could have possibly contributed to the suicide and stated ‘we were also in turmoil…our marriage was falling apart. Could I have done something?’…

(Link et al. [40, 68])

Participants described how, of the individuals they had known who had died by suicide, in many cases the person had experienced recent relationship difficulties or a separation/divorce from their partner, and this was particularly the case for male farmers.

(Perceval et al. [13, p. 4])

The quality and stability of spousal relationships may also have implications for self‐harm but were not explicitly covered in the included papers.

#### 3.5.2 Perceptions of Self‐Harm and Help‐Seeking (High Confidence) [1–5, 11, and 14]

This subtheme describes the views and responses to people who self‐harm and mental ill‐health, more broadly, among people in rural communities; this revealed important cultural values which may contribute to self‐harm. It was noted that those in some contexts, for example, Aboriginal communities, members would avoid discussing trauma and negative emotions:

…the huge emotional burden that one has to carry from childhood on account of not being able to share traumatic experiences is a factor that contributes to suicide and self‐harm in young people and adults.

(Issacs et al. [41, 172])

A study from Ethiopia also described reluctance within communities to acknowledge and or support people who attempted suicide:

…it is unusual for suicide attempters to be taken to clinics. Such action may invite unwanted attention.

(Jacobsson et al. [42, 67])

Avoidance of publicly acknowledging self‐harm within rural communities seems apparently stable over different cultural contexts and times.

Gendered norms were also highlighted as a means through which to silence accounts of self‐harm and mental ill‐health. Masculine identities embedded in hegemony and stoicism were described in the evidence:

Participants also described how sharing problems with peers was considered to be a sign of weakness particularly among men.

(Isaacs et al. [41, 172])

Most participants spoke about stigma in mental illness and suggested that the men lost to suicide may have thought that revealing their depression would be interpreted by others as weakness.

(Creighton et al. [43, 4])

These statements and others [13, p. 283] (Creighton et al. 2017, 6) describe pressures and expectations on men in rural communities to conform to strong and silent identities or risk being associated as ‘weak’ among peers. This had clear implications for help‐seeking; Perceval et al. state

Negative community attitudes and stigma towards mental health issues and suicide were cited by participants as an issue for farmers and those living in rural areas. Despite recent efforts within the suicide prevention and mental health spaces in rural areas… the fear of having others in the community talk negatively and subsequently feeling like a failure was described as a compelling barrier to help seeking.

(Perceval et al. [13, p. 3])

### 3.6 Accessing Healthcare in Rural Areas

This theme summarises evidence on the perceptions among healthcare professionals in rural communities about self‐harm. The theme also highlights limited opportunities for professional development and access to resources in these areas.

#### 3.6.1 Lack of Awareness and Training (Moderate to High Confidence) [1, 2, 6, 7, 9, 10, 13, and 14]

Judgemental attitudes and a lack of recognition of responsibility among healthcare providers about self‐harm and people who self‐harm were described in several papers (Slaven and Kisely 2002; Fitzpatrick et al. 2021) [16, 17, 44, 45, 46]; this presented a barrier for people accessing healthcare:

A barrier cited by all Mental Health participants was the display of judgmental or indifferent attitudes by some [emergency department] hospital staff towards DSH [Deliberate self‐harm] patients. [Mental Health Practitioners] also felt that hospital staff did not perceive the assessment or management of DSH patients being their responsibility.

(Slaven and Kisely [44, 236] [text added not in original])

A lack of awareness and training around mental ill‐health was also apparent across a variety of different countries, for example, from Sri Lanka and Western Australia; this highlighted a lack of access to relevant professional education and training in these rural areas:

Meanwhile others interviewed pointed to the challenges of continuing professional educations (CPE) in rural hospital settings and how such challenges can limit the effectiveness of care for the self‐poisoning patient.

(Senarathna et al. [16, p. 5])

The nursing staff don't know what to do with them, you feel inadequate. We aren't trained to deal with mental health issues.

(Slaven and Kisely [44, 235])

#### 3.6.2 Lack of Support and Resources (Moderate to High Confidence) [1–14]

Formal and informal sources of support were reported to be in short supply in rural areas for people who self‐harmed; key infrastructural barriers were also highlighted that prevented individuals from accessing appropriate healthcare services. A lack of formal support for people in rural areas influenced attitudes towards help‐seeking:

…if they want to schedule appointments with mental health providers, it's like, well, this is 2 months in advance. So, really disheartening to wanna reach out and you're willing to get help, but just can't get it.

(George et al. [15, p. 6])

… the sample were fortunate that they had access to reasonable medical care…. but the researchers have no doubt that some of our sample would not have survived if they had been living in even more remote areas.

(Pearson et al. [26, p. 364])

It was also reported that rural areas have limited infrastructure in terms of healthcare services and more general amenities, which again influenced decisions to seek help:

Participants commonly noted how a lack of public transportation also resulted in limited access to healthcare or mental health resources.

(George et al. [15, p. 6])

The isolation was further complicated by poor access to telecommunications, such as mobile phone coverage and internet availability.

(Perceval et al. [13, p. 281])

These data acknowledge how people that live in rural areas, particularly deprived and remote areas, may face material barriers to help‐seeking that go beyond attitudes and awareness. Without access to transport or telecommunications for remote treatment or support takes away the opportunity for those in these communities to get the help and support they need.

## 4 Discussion

### 4.1 Summary of Findings

This review synthesised findings from 14 qualitative and mixed‐methods studies on self‐harm in rural environments. The included papers provided a global perspective on this topic with papers from the United States, Australia, Sri Lanka and China. Two analytical themes were identified: (1) Experiences of self‐harm that describe reasons for self‐harm, community‐held perceptions about self‐harm, perceptions and (2) accessing healthcare in rural areas, which discussed responses to self‐harm by rural healthcare providers, and access to healthcare services. We identified with high confidence that reasons for self‐harm were linked to agricultural occupations and area‐level deprivation and decline. Further, agricultural occupations were linked to loneliness and isolation, which contributed to poor mental health. Relationship difficulties and comorbid physical health problems were also revealed as contributing to self‐harm behaviour. We also identified with high confidence that perceptions of self‐harm were shaped by culture and often shrouded in stigma and hegemonic, potentially toxic, forms of masculinity; agricultural occupations were commonly foregrounded. We identified with moderate to high confidence that healthcare providers in some rural areas held negative views of people who self‐harm and lacked adequate training on mental health. We also identified, with moderate to high confidence, that people living in rural areas experienced limited access to healthcare services due to a lack of local services or poor transport and telecommunication networks, which were all barriers to help‐seeking. Overall, there was an indication of high strength within the evidence, as demonstrated by moderate to high confidence levels in the synthesis.

### 4.2 Comparison With Existing Literature

Aligning with findings by Purc‐Stephenson et al. [23], our synthesised findings also suggest that reasons for self‐harm among people in rural areas may be linked *directly* to characteristics of these areas, such as agricultural occupations and social isolation. Similarly, previous research on loneliness has revealed similar links to rurality and farming [47]. Living with physical comorbidities [23, 48] and marital distress were also identified in this study. Marital problems, including domestic violence, were also identified as a contributing factor to self‐harm and self‐harm attitudes [49]. Marital struggles could also be linked with cultural attitudes and stigma towards divorce [50, 51]. Subsequently, people may stay in unhealthy or abusive relationships that negatively affect their mental health [52, 53].

The review findings indicate that isolation has been identified as a key predictor of self‐harm in rural communities [7, 8, 9, 54]. Findings also revealed that isolation due to geographical location could be a contributing factor to self‐harm. It was widely discussed across the included evidence that the nature of work in rural areas (e.g., farming) and corresponding isolation and loneliness were detrimental to a person's mental health and could have been a cause for suicide, and self‐harm [23, 25]. Although some factors such as isolation and marital difficulties are not specific to these communities, they may be exacerbated by living in rural areas, due to geographical location, poor communication and transport links and rural‐specific careers [14, 55, 56].

Negative community attitudes and stigma were also highlighted in the included papers: this relates to notions such as gender disparity, with the suggestion that men within rural communities are more at risk due to gender‐specific stigma as well as behavioural expectations that are specific to their more stereotypical masculine roles. These findings can be supported by previous research that indicates that mental illness stigma is more prominent in males compared to females in rural communities [57]. The findings also provided insight into negative community attitudes in relation to cultural stigma in countries such as Ethiopia and within Aboriginal communities.

A lack of adequate resources and training in mental health, specifically self‐harm, among healthcare professionals in rural communities has been reported elsewhere [58]. This education disparity may explain the stigmatised and judgemental approaches by healthcare staff that were highlighted in some of the studies [59]. Further, the lack of awareness and training may have contributed to the conflation of self‐harm and suicidal behaviour, as has been reported previously in relation to general practitioners [60].

### 4.3 Strengths and Limitations

The synthesis provides a novel summary of published qualitative evidence on self‐harm in global rural contexts. The qualitative studies included in this review relate predominantly to self‐harm leading to suicide (or self‐harm with intent) rather than NSSI or poisoning. However, findings help to identify important aspects of self‐harm with intent in rural settings, such as, social and healthcare perspectives. Commonalities in experiences and observations were shared despite geographical variation between the included studies. Views of people with lived experience of self‐harm (personal and as a carer) were sought on the study design and preliminary findings, via a PPIE group. This enhanced the study design and improved credibility.

An initial scope of the literature suggested that the body of literature on rural self‐harm was small. We anticipated that some data relevant to rural self‐harm may be contained within papers on rural suicide. We therefore adopted an inclusive approach to the selection of papers to generate a larger pool for full‐text reviews, before making a final decision on inclusion/exclusion. This approach maximised the number of included papers. However, for papers that primarily focused on rural suicide, the difference between self‐harm and suicide was often unclear and at times conflated. Data specifically on self‐harm were, therefore, difficult to disentangle and extract; this is a limitation of our review and this field of research. Future studies need to be more explicit in definitions and to explore self‐harm as an individual phenomenon separate from suicide.

### 4.4 Implications for Practice and Policy

This rapid review highlights a scarcity in research that specifically focuses on rural self‐harm; this stands in stark contrast to the abundance of published evidence exploring rural suicide. If public health interventions are to succeed in preventing suicide, then self‐harm should be more widely considered and targeted. To do this, further research is needed to understand the different forms and functions of rural self‐harm.

Rural areas experience disparities in access to healthcare services either directly as a result of services being located in urban centres with higher population densities or as a consequence of poor transport or telecommunications infrastructure. Alternative ways of accessing healthcare for people in these areas who self‐harm may be required (e.g., outreach services) and or better training for rural healthcare practitioners to support disclosure. Voluntary sector organisations may provide critical capacity in this space and help to reduce stigma around mental ill‐health.

## 5 Conclusion

The overall aims for the review were (1) to synthesise current qualitative evidence about experiences of self‐harm among adults living in rural areas, including formal and informal care provider perspectives, and to identify social and environmental factors that influence access to care. (2) Identify gaps in knowledge and opportunities for future interventions for rural adults at risk of self‐harm in an underserved community. The current study provides evidence of the challenges relating to unpicking data from studies that relate to suicide and suicidal behaviour, when exploring self‐harm due to these notions typically being used interchangeably.

Reasons for self‐harm were reported such as isolation, access to means and relationship issues. Cultural differences were also noted, from not only within farming communities, gender disparity but also within religious and spiritual cultures. Evidence also suggests that there is limited access to mental health support services in rural communities, and healthcare professionals feel they are not trained to support those with mental health difficulties. Finally, findings provide a gap in research for qualitative exploration of self‐harm within rural communities in the United Kingdom due to the lack of research currently in this area.

## Author Contributions

**Katie Saunders:** data curation, investigation, visualization, writing – original draft, writing – review and editing, formal analysis, project administration. **William Nicholls:** conceptualization, methodology, data curation, investigation, validation, funding acquisition, writing – review and editing. **Nadia Corp:** conceptualization, methodology, data curation, supervision, funding acquisition, visualization, writing – review and editing. **Tom Kingstone:** conceptualization, methodology, data curation, investigation, validation, formal analysis, supervision, funding acquisition, visualization, project administration, writing – review and editing. **Faraz Mughal:** conceptualization, methodology, supervision, investigation, funding acquisition, visualization, writing – review and editing. **Carolyn A. Chew‐Graham:** conceptualization, funding acquisition, visualization, writing – review and editing, methodology, investigation. **Jane Southam:** conceptualization, funding acquisition, visualization, writing – review and editing. **Tamsin Fisher:** conceptualization, methodology, data curation, investigation, validation, formal analysis, supervision, funding acquisition, visualization, project administration, writing – review and editing.

## Ethics Statement

The authors have nothing to report.

## Conflicts of Interest

C.A.C.‐G. is Editor in Chief of *HEX*. T.K. is a member of the Editorial Board of *HEX*.

## Supporting information

FILE 001 Experiences and perceptions of self‐harm in rural‐dwelling adults A rapid review of qualitative evidence SEARCHES.

FILE 002_Experiences and perceptions of self‐harm in rural‐dwelling adults _A rapid review of qualitative evidence w_Supplimentary tables.

## Data Availability

The authors have nothing to report.

## Endnotes

1 Study was excluded before analysis.
