# Supplementary material for: Experiences and Perceptions of Self‐Harm in Rural‐Dwelling Adults: A Rapid Review of Qualitative Evidence
Source: Health Expect. 2025 Apr 25;28(3):e70268. doi: 10.1111/hex.70268 (PMC12022502; doi:10.1111/hex.70268)
Supplement: Supplementary file 1 — FILE 001 Experiences and perceptions of self‐harm in rural‐dwelling adults A rapid review of qualitative evidence SEARCHES. [file HEX-28-e70268-s001.docx]

## Experiences of self-harm among rural adults: Rapid Review

All searches conducted 26.06.2023

## Search strategies

The following table is an explanation of the symbols used in the search strategies below.

| / | indicates an index term (MeSH heading) (Ovid) |
| --- | --- |
| exp | before an index term indicates that all subheadings were selected (Ovid) |
| AB | indicates a search for a term in the abstract (EBSCO, WoS) |
| AK | indicates a search for a term in the author keywords (WoS) |
| DE | indicates an index term (EBSCO: PsycINFO) |
| MH | indicates an index term (EBSCO: CINAHL) |
| .mp | indicates a search for a term in multiple purpose included, title, abstract, subject heading word, floating sub-heading word, keyword heading word (MEDLINE/EMBASE) |
| TI | indicates a search for a term in the title (EBSCO, WoS) |
| .ti,ab,kf | indicates a search for a term in the title/abstract/word(s) in keyword (MEDLINE/EMBASE) |
| .tw | indicates a search for a term in the title/abstract (MEDLINE/EMBASE) |
| * | at the end of a term indicates that this term has been truncated. |
| # | Mandated wild card character stands for one character within, or at the end of, a word. (Ovid) |
| ? | Optional wild card character stands for zero or one character within, or at the end of, a word (Ovid) |
| adj | Search for two terms where they appear adjacent to each another in the same order (Ovid) |
| adj*n* | Search for two terms where they appear within *n* words of each another. (Ovid) |
| N*n* | Search for two terms where they appear within *n*+1 words of each another. (EBSCO) |
| near/*n* | Search for two terms where they appear within *n* words of each another. (Web of Science, Proquest: ASSIA) |
| Pre/*n* | Search for two terms where they appear within *n* words of each another in exact order. (Proquest: ASSIA) |
| W*n* | Search for two terms where they appear within *n*+1 words of each another in exact order. (EBSCO) |
|  |  |
|  |  |

## MEDLINE (OvidSP) - Ovid MEDLINE(R) ALL 1946 to June 22, 2023

| 1 | exp Self-Injurious Behavior/ |
| --- | --- |
| 2 | exp Drug Overdose/ |
| 3 | ((self or themsel* or himsel* or hersel*) adj3 harm*).ti,ab,kf. |
| 4 | ((self or themsel* or himsel* or hersel*) adj3 mutilat*).ti,ab,kf. |
| 5 | ((self or themsel* or himsel* or hersel*) adj3 injur*).ti,ab,kf. |
| 6 | ((self or themsel* or himsel* or hersel*) adj3 poison*).ti,ab,kf. |
| 7 | ((self or themsel* or himsel* or hersel*) adj3 cut*).ti,ab,kf. |
| 8 | ((self or themsel* or himsel* or hersel*) adj3 inflict*).ti,ab,kf. |
| 9 | ((self or themsel* or himsel* or hersel*) adj3 destruct*).ti,ab,kf. |
| 10 | ((self or themsel* or himsel* or hersel*) adj3 wound*).ti,ab,kf. |
| 11 | ((self or themsel* or himsel* or hersel*) adj3 violen*).ti,ab,kf. |
| 12 | (auto mutilat* or automutilat*).ti,ab,kf. |
| 13 | overdos*.ti,ab,kf. |
| 14 | over dos*.ti,ab,kf. |
| 15 | headbang*.ti,ab,kf. |
| 16 | head bang*.ti,ab,kf. |
| 17 | parasuicid*.ti,ab,kf. |
| 18 | para suicid*.ti,ab,kf. |
| 19 | (suicid* adj3 (ideation* or attempt*)).ti,ab,kf. |
| 20 | or/1-19 |
| 21 | Rural Health/ |
| 22 | Rural Population/ |
| 23 | rural*.ti,ab,kf. |
| 24 | countryside.ti,ab,kf. |
| 25 | country side.ti,ab,kf. |
| 26 | nonurban*.ti,ab,kf. |
| 27 | non urban*.ti,ab,kf. |
| 28 | remote*.ti,ab,kf. |
| 29 | village*.ti,ab,kf. |
| 30 | Agriculture/ |
| 31 | agricultur*.ti,ab,kf. |
| 32 | husbandry.ti,ab,kf. |
| 33 | Farmers/ |
| 34 | farm*.ti,ab,kf. |
| 35 | (veterinarian or vet or vets or (veterinary adj (surgeon* or nurse* or practitioner*))).ti,ab,kf. |
| 36 | or/21-35 |
| 37 | qualitative research/ [After DeJean et al., 2016. Qual Health Res 26(10): 1307-1317] |
| 38 | interview/ |
| 39 | nursing methodology research/ |
| 40 | (theme$ or thematic).mp. |
| 41 | qualitative.af. |
| 42 | questionnaire$.mp. |
| 43 | ethnological research.mp. |
| 44 | ethnograph$.mp. |
| 45 | ethnonursing.af. |
| 46 | phenomenol$.af. |
| 47 | (grounded adj (theor$ or study or studies or research or analys?s)).af. |
| 48 | (life stor$ or women$ stor$).mp. |
| 49 | (emic or etic or hermeneutic$ or heuristic$ or semiotic$).af. |
| 50 | ((data adj1 saturat$) or participant observ$).tw. |
| 51 | (social construct$ or postmodern$ or post modern$ or poststructural$ or post structural$ or feminis$ or interpret$).mp. |
| 52 | (action research or cooperative inquir$ or co operative inquir$).mp. |
| 53 | (humanistic or existential or experiential or paradigm$).mp. |
| 54 | (field adj (study or studies or research)).tw. |
| 55 | human science.tw. |
| 56 | biographical method.tw. |
| 57 | theoretical sampl$.af. |
| 58 | ((purpos$ adj4 sampl$) or (focus adj group$)).af. |
| 59 | (account or accounts or unstructured or open ended or text$ or narrative$).mp. |
| 60 | (life world or conversation analys?s or personal experience$ or theoretical saturation).mp. |
| 61 | ((lived or life) adj experience$).mp. |
| 62 | cluster sampl$.mp. |
| 63 | observational method$.af. |
| 64 | content analysis.af. |
| 65 | (constant adj (comparative or comparison)).af. |
| 66 | ((discourse$ or discurs$) adj3 analys?s).tw. |
| 67 | narrative analys?s.af. |
| 68 | heidegger$.tw. |
| 69 | colaizzi$.tw. |
| 70 | spiegelberg$.tw. |
| 71 | van manen$.tw. |
| 72 | van kaam$.tw. |
| 73 | merleau ponty$.tw. |
| 74 | husserl$.tw. |
| 75 | foucault$.tw. |
| 76 | (corbin$ adj2 strauss$).tw. |
| 77 | glaser$.tw. |
| 78 | (mix$ adj2 (method$ or design$)).af. [filter amended to identify mixed method studies] |
| 79 | or/37-78 |
| 80 | 20 and 36 and 79 |

## EMBASE (OvidSP) - Embase 1974 to 2023 June 23

| 1 | automutilation/ |
| --- | --- |
| 2 | exp drug Overdose/ |
| 3 | ((self or themsel* or himsel* or hersel*) adj3 harm*).ti,ab,kf. |
| 4 | ((self or themsel* or himsel* or hersel*) adj3 mutilat*).ti,ab,kf. |
| 5 | ((self or themsel* or himsel* or hersel*) adj3 injur*).ti,ab,kf. |
| 6 | ((self or themsel* or himsel* or hersel*) adj3 poison*).ti,ab,kf. |
| 7 | ((self or themsel* or himsel* or hersel*) adj3 cut*).ti,ab,kf. |
| 8 | ((self or themsel* or himsel* or hersel*) adj3 inflict*).ti,ab,kf. |
| 9 | ((self or themsel* or himsel* or hersel*) adj3 destruct*).ti,ab,kf. |
| 10 | ((self or themsel* or himsel* or hersel*) adj3 wound*).ti,ab,kf. |
| 11 | ((self or themsel* or himsel* or hersel*) adj3 violen*).ti,ab,kf. |
| 12 | (auto mutilat* or automutilat*).ti,ab,kf. |
| 13 | overdos*.ti,ab,kf. |
| 14 | over dos*.ti,ab,kf. |
| 15 | headbang*.ti,ab,kf. |
| 16 | head bang*.ti,ab,kf. |
| 17 | parasuicid*.ti,ab,kf. |
| 18 | para suicid*.ti,ab,kf. |
| 19 | (suicid* adj3 (ideation* or attempt*)).ti,ab,kf. |
| 20 | or/1-19 |
| 21 | rural health/ |
| 22 | exp rural health care/ |
| 23 | rural population/ |
| 24 | rural area/ |
| 25 | rural hospital/ |
| 26 | rural*.ti,ab,kf. |
| 27 | countryside.ti,ab,kf. |
| 28 | country side.ti,ab,kf. |
| 29 | nonurban*.ti,ab,kf. |
| 30 | non urban*.ti,ab,kf. |
| 31 | remote*.ti,ab,kf. |
| 32 | village*.ti,ab,kf. |
| 33 | exp agriculture/ |
| 34 | husbandry.ti,ab,kf. |
| 35 | agricultur*.ti,ab,kf. |
| 36 | agricultural worker/ |
| 37 | farm*.ti,ab,kf. |
| 38 | exp veterinarian/ |
| 39 | (veterinarian or vet or vets or (veterinary adj (surgeon* or nurse* or practitioner*))).ti,ab,kf. |
| 40 | or/21-39 |
| 41 | qualitative research/ |
| 42 | exp interview/ |
| 43 | nursing methodology research/ |
| 44 | (theme$ or thematic).mp. |
| 45 | qualitative.af. |
| 46 | questionnaire$.mp. |
| 47 | ethnological research.mp. |
| 48 | ethnograph$.mp. |
| 49 | ethnonursing.af. |
| 50 | phenomenol$.af. |
| 51 | (grounded adj (theor$ or study or studies or research or analys?s)).af. |
| 52 | (life stor$ or women$ stor$).mp. |
| 53 | (emic or etic or hermeneutic$ or heuristic$ or semiotic$).af. |
| 54 | ((data adj1 saturat$) or participant observ$).tw. |
| 55 | (social construct$ or postmodern$ or post modern$ or poststructural$ or post structural$ or feminis$ or interpret$).mp. |
| 56 | (action research or cooperative inquir$ or co operative inquir$).mp. |
| 57 | (humanistic or existential or experiential or paradigm$).mp. |
| 58 | (field adj (study or studies or research)).tw. |
| 59 | human science.tw. |
| 60 | biographical method.tw. |
| 61 | theoretical sampl$.af. |
| 62 | ((purpos$ adj4 sampl$) or (focus adj group$)).af. |
| 63 | (account or accounts or unstructured or open ended or text$ or narrative$).mp. |
| 64 | (life world or conversation analys?s or personal experience$ or theoretical saturation).mp. |
| 65 | ((lived or life) adj experience$).mp. |
| 66 | cluster sampl$.mp. |
| 67 | observational method$.af. |
| 68 | content analysis.af. |
| 69 | (constant adj (comparative or comparison)).af. |
| 70 | ((discourse$ or discurs$) adj3 analys?s).tw. |
| 71 | narrative analys?s.af. |
| 72 | heidegger$.tw. |
| 73 | colaizzi$.tw. |
| 74 | spiegelberg$.tw. |
| 75 | van manen$.tw. |
| 76 | van kaam$.tw. |
| 77 | merleau ponty$.tw. |
| 78 | husserl$.tw. |
| 79 | foucault$.tw. |
| 80 | (corbin$ adj2 strauss$).tw. |
| 81 | glaser$.tw. |
| 82 | or/41-81 |
| 83 | 20 and 40 and 82 |
| 84 | limit 83 to embase |

## CINAHLPlus (EBSCO)

| S1 | (MH "Injuries, Self-Inflicted") |
| --- | --- |
| S2 | (MH "Overdose+") |
| S3 | TI ( (self OR themsel* OR himsel* OR hersel*) N2 harm* ) OR AB ( (self OR themsel* OR himsel* OR hersel*) N2 harm* ) |
| S4 | TI ( (self OR themsel* OR himsel* OR hersel*) N2 mutilat* ) OR AB ( (self OR themsel* OR himsel* OR hersel*) N2 mutilat* ) |
| S5 | TI ( (self OR themsel* OR himsel* OR hersel*) N2 injur* ) OR AB ( (self OR themsel* OR himsel* OR hersel*) N2 injur* ) |
| S6 | TI ( (self OR themsel* OR himsel* OR hersel*) N2 poison* ) OR AB ( (self OR themsel* OR himsel* OR hersel*) N2 poison* ) |
| S7 | TI ( (self OR themsel* OR himsel* OR hersel*) N2 cut* ) OR AB ( (self OR themsel* OR himsel* OR hersel*) N2 cut* ) |
| S8 | TI ( (self OR themsel* OR himsel* OR hersel*) N2 inflict* ) OR AB ( (self OR themsel* OR himsel* OR hersel*) N2 inflict* ) |
| S9 | TI ( (self OR themsel* OR himsel* OR hersel*) N2 destruct* ) OR AB ( (self OR themsel* OR himsel* OR hersel*) N2 destruct* ) |
| S10 | TI ( (self OR themsel* OR himsel* OR hersel*) N2 wound* ) OR AB ( (self OR themsel* OR himsel* OR hersel*) N2 wound* ) |
| S11 | TI ( (self OR themsel* OR himsel* OR hersel*) N2 violen* ) OR AB ( (self OR themsel* OR himsel* OR hersel*) N2 violen* ) |
| S12 | TI ( (auto W0 mutilat*) OR automutilat* ) OR AB ( (auto W0 mutilat*) OR automutilat* ) |
| S13 | TI overdos* OR AB overdos* |
| S14 | TI over W0 dos* OR AB over W0 dos* |
| S15 | TI headbang* OR AB headbang* |
| S16 | TI head W0 bang* OR AB head W0 bang* |
| S17 | TI parasuicid* OR AB parasuicid* |
| S18 | TI para W0 suicid* OR AB para W0 suicid* |
| S19 | TI ( suicid* N2 (ideation* OR attempt*) ) OR AB ( suicid* N2 (ideation* OR attempt*) ) |
| S20 | S1 OR S2 OR S3 OR S4 OR S5 OR S6 OR S7 OR S8 OR S9 OR S10 OR S11 OR S12 OR S13 OR S14 OR S15 OR S16 OR S17 OR S18 OR S19 |
| S21 | (MH "Rural Population") OR (MH "Rural Areas") |
| S22 | (MH "Rural Health Personnel") OR (MH "Rural Health Centers") OR (MH "Hospitals, Rural") OR (MH "Rural Nurses") OR (MH "Rural Health Services") OR (MH "Rural Nursing") OR (MH "Rural Health") OR (MH "Australian Rural Nurses and Midwives") OR (MH "Association for Australian Rural Nurses") OR (MH "Services for Australian Rural and Remote Allied Health") |
| S23 | TI rural* OR AB rural* |
| S24 | TI countryside OR AB countryside |
| S25 | TI country W0 side OR AB country W0 side |
| S26 | TI nonurban* OR AB nonurban* |
| S27 | TI non W0 urban* OR AB non W0 urban* |
| S28 | TI remote* OR AB remote* |
| S29 | TI village* OR AB village* |
| S30 | (MH "Agriculture+") |
| S31 | TI agricultur* OR AB agricultur* |
| S32 | TI husbandry OR AB husbandry |
| S33 | (MH "Farmworkers") |
| S34 | TI farm* OR AB farm* |
| S35 | TI ( (veterinarian OR vet OR vets OR (veterinary W0 (surgeon* OR nurse* OR practitioner*)) ) OR AB ( (veterinarian OR vet OR vets OR (veterinary W0 (surgeon* OR nurse* OR practitioner*)) ) |
| S36 | S21 OR S22 OR S23 OR S24 OR S25 OR S26 OR S27 OR S28 OR S29 OR S30 OR S31 OR S32 OR S33 OR S34 OR S35 |
| S37 | MH Interview+ |
| S38 | MH audiorecording |
| S39 | MH interviews |
| S40 | MH Grounded theory |
| S41 | MH Qualitative Studies |
| S42 | MH Research, Nursing |
| S43 | MH Questionnaires+ |
| S44 | MH Focus Groups |
| S45 | MH Discourse Analysis |
| S46 | MH Content Analysis |
| S47 | MH Ethnographic Research |
| S48 | MH Ethnological Research |
| S49 | MH Ethnonursing Research |
| S50 | MH Constant Comparative Method |
| S51 | MH Qualitative Validity+ |
| S52 | MH Purposive Sample |
| S53 | MH Observational Methods+ |
| S54 | MH Field Studies |
| S55 | MH theoretical sample |
| S56 | MH Phenomenology |
| S57 | MH Phenomenological Research |
| S58 | MH Life Experiences+ |
| S59 | MH Cluster Sample+ |
| S60 | ethnonursing |
| S61 | ethnograph* |
| S62 | phenomenol* |
| S63 | grounded W0 theor* |
| S64 | "grounded study" |
| S65 | "grounded studies" |
| S66 | "grounded research" |
| S67 | grounded W0 analys?s |
| S68 | life W0 stor* |
| S69 | women's W0 stor* |
| S70 | emic or etic or hermeneutic* or heuristic* or semiotic* |
| S71 | data N0 saturat* |
| S72 | participant W0 observ* |
| S73 | social W0 construct* OR post-modern* OR post-structural* OR poststructural* OR postmodern* OR feminis* OR interpret* |
| S74 | "action research" OR cooperative W0 inquir* OR co-operative W0 inquir* |
| S75 | humanistic OR existential OR experiental OR paradigm* |
| S76 | field W0 stud* |
| S77 | "field research" |
| S78 | "human science" |
| S79 | "biographical method" |
| S80 | theoretical W0 sampl* |
| S81 | purpos* N3 sampl* |
| S82 | focus W0 group* |
| S83 | account OR accounts OR unstructured OR open-ended OR text* OR narrative* |
| S84 | life-world OR conversation W0 analys?s OR personal W0 experience* OR theoretical W0 saturation |
| S85 | lived W0 experience* |
| S86 | life W0 experience* |
| S87 | cluster W0 sampl* |
| S88 | theme* OR thematic |
| S89 | observational W0 method* |
| S90 | questionnaire* |
| S91 | "content analysis" |
| S92 | discourse* N2 analys?s |
| S93 | discurs* N2 analys?s |
| S94 | "constant comparative" |
| S95 | "constant comparison" |
| S96 | "narrative analysis" |
| S97 | Heidegger* |
| S98 | Colaizzi* |
| S99 | Spiegelberg* |
| S100 | van W0 manen* |
| S101 | van W0 kaam* |
| S102 | merleau W0 ponty* |
| S103 | husserl* |
| S104 | Foucault* |
| S105 | corbin* N1 strauss* |
| S106 | glaser* |
| S107 | S37 OR S38 OR S39 OR S40 OR S41 OR S42 OR S43 OR S44 OR S45 OR S46 OR S47 OR S48 OR S49 OR S50 OR S51 OR S52 OR S53 OR S54 OR S55 OR S56 OR S57 OR S58 OR S59 OR S60 OR S61 OR S62 OR S63 OR S64 OR S65 OR S66 OR S67 OR S68 OR S69 OR S70 OR S71 OR S72 OR S73 OR S74 OR S75 OR S76 OR S77 OR S78 OR S79 OR S80 OR S81 OR S82 OR S83 OR S84 OR S85 OR S86 OR S87 OR S88 OR S89 OR S90 OR S91 OR S92 OR S93 OR S94 OR S95 OR S96 OR S97 OR S98 OR S99 OR S100 OR S101 OR S102 OR S103 OR S104 OR S105 OR S106 |
| S108 | S20 AND S36 AND S107 |

## PsycINFO (EBSCO)

| S1 | DE "Nonsuicidal Self-Injury" OR DE "Head Banging" OR DE "Self-Inflicted Wounds" OR DE "Self-Poisoning" OR DE "Attempted Suicide" |
| --- | --- |
| S2 | DE "Drug Overdoses" |
| S3 | TI ( (self OR themsel* OR himsel* OR hersel*) N2 harm* ) OR AB ( (self OR themsel* OR himsel* OR hersel*) N2 harm* ) |
| S4 | TI ( (self OR themsel* OR himsel* OR hersel*) N2 mutilat* ) OR AB ( (self OR themsel* OR himsel* OR hersel*) N2 mutilat* ) |
| S5 | TI ( (self OR themsel* OR himsel* OR hersel*) N2 injur* ) OR AB ( (self OR themsel* OR himsel* OR hersel*) N2 injur* ) |
| S6 | TI ( (self OR themsel* OR himsel* OR hersel*) N2 poison* ) OR AB ( (self OR themsel* OR himsel* OR hersel*) N2 poison* ) |
| S7 | TI ( (self OR themsel* OR himsel* OR hersel*) N2 cut* ) OR AB ( (self OR themsel* OR himsel* OR hersel*) N2 cut* ) |
| S8 | TI ( (self OR themsel* OR himsel* OR hersel*) N2 inflict* ) OR AB ( (self OR themsel* OR himsel* OR hersel*) N2 inflict* ) |
| S9 | TI ( (self OR themsel* OR himsel* OR hersel*) N2 destruct* ) OR AB ( (self OR themsel* OR himsel* OR hersel*) N2 destruct* ) |
| S10 | TI ( (self OR themsel* OR himsel* OR hersel*) N2 wound* ) OR AB ( (self OR themsel* OR himsel* OR hersel*) N2 wound* ) |
| S11 | TI ( (self OR themsel* OR himsel* OR hersel*) N2 violen* ) OR AB ( (self OR themsel* OR himsel* OR hersel*) N2 violen* ) |
| S12 | TI ( (auto W0 mutilat*) OR automutilat* ) OR AB ( (auto W0 mutilat*) OR automutilat* ) |
| S13 | TI overdos* OR AB overdos* |
| S14 | TI over W0 dos* OR AB over W0 dos* |
| S15 | TI headbang* OR AB headbang* |
| S16 | TI head W0 bang* OR AB head W0 bang* |
| S17 | TI parasuicid* OR AB parasuicid* |
| S18 | TI para W0 suicid* OR AB para W0 suicid* |
| S19 | TI ( suicid* N2 (ideation* OR attempt*) ) OR AB ( suicid* N2 (ideation* OR attempt*) ) |
| S20 | S1 OR S2 OR S3 OR S4 OR S5 OR S6 OR S7 OR S8 OR S9 OR S10 OR S11 OR S12 OR S13 OR S14 OR S15 OR S16 OR S17 OR S18 OR S19 |
| S21 | DE "Rural Environments" |
| S22 | DE "Rural Health" |
| S23 | TI rural* OR AB rural* |
| S24 | TI countryside OR AB countryside |
| S25 | TI country W0 side OR AB country W0 side |
| S26 | TI nonurban* OR AB nonurban* |
| S27 | TI non W0 urban* OR AB non W0 urban* |
| S28 | TI remote* OR AB remote* |
| S29 | TI village* OR AB village* |
| S30 | DE "Agriculture" |
| S31 | TI agricultur* OR AB agricultur* |
| S32 | TI husbandry OR AB husbandry |
| S33 | DE "Agricultural Workers" OR DE "Migrant Farm Workers" OR DE "Agricultural Extension Workers" |
| S34 | TI farm* OR AB farm* |
| S35 | TI ( (veterinarian OR vet OR vets OR (veterinary W0 (surgeon* OR nurse* OR practitioner*)) ) OR AB ( (veterinarian OR vet OR vets OR (veterinary W0 (surgeon* OR nurse* OR practitioner*)) ) |
| S36 | S21 OR S22 OR S23 OR S24 OR S25 OR S26 OR S27 OR S28 OR S29 OR S30 OR S31 OR S32 OR S33 OR S34 OR S35 |
| S37 | DE "Interviewing" |
| S38 | DE "Interviews" OR DE "Intake Interview" OR DE "Interview Schedules" OR DE "Job Applicant Interviews" OR DE "Psychodiagnostic Interview" |
| S39 | DE "Grounded Theory" |
| S40 | DE "Qualitative Research" |
| S41 | DE "Questionnaires" OR DE "General Health Questionnaire" |
| S42 | DE "Discourse Analysis" |
| S43 | DE "Content Analysis" |
| S44 | DE "Observation Methods" |
| S45 | DE "Phenomenology" |
| S46 | DE "Life Experiences" OR DE "Life Changes" |
| S47 | ethnonursing |
| S48 | ethnograph* |
| S49 | phenomenol* |
| S50 | grounded W0 theor* |
| S51 | "grounded study" |
| S52 | "grounded studies" |
| S53 | "grounded research" |
| S54 | grounded W0 analys?s |
| S55 | life W0 stor* |
| S56 | women's W0 stor* |
| S57 | emic or etic or hermeneutic* or heuristic* or semiotic* |
| S58 | data W0 saturat* |
| S59 | participant W0 observ* |
| S60 | social W0 construct* OR post-modern OR post-structural* OR poststructural* OR postmodern* OR feminis* OR interpret* |
| S61 | "action research" OR cooperative W0 inquir* OR co-operative inquir* |
| S62 | humanistic OR existential OR experiental OR paradigm* |
| S63 | field W0 stud* |
| S64 | "field research" |
| S65 | "human science" |
| S66 | "biographical method" |
| S67 | theoretical W0 sampl* |
| S68 | purpos* N3 sampl* |
| S69 | focus W0 group* |
| S70 | account OR accounts OR unstructured OR open-ended OR text* OR narrative* |
| S71 | life-world OR conversation W0 analys?s OR personal W0 experience* OR theoretical W0 saturation |
| S72 | lived W0 experience* |
| S73 | life W0 experience* |
| S74 | cluster W0 sampl* |
| S75 | theme* OR thematic |
| S76 | observational W0 method* |
| S77 | questionnaire* |
| S78 | "content analysis" |
| S79 | discourse* N2 analys?s |
| S80 | discurs* N2 analys?s |
| S81 | "constant comparative" |
| S82 | "constant comparison" |
| S83 | narrative analysis |
| S84 | Heidegger* |
| S85 | Colaizzi* |
| S86 | Spiegelberg* |
| S87 | van W0 manen* |
| S88 | van W0 kaam* |
| S89 | merleau W0 ponty* |
| S90 | husserl* |
| S91 | Foucault* |
| S92 | corbin* N1 strauss* |
| S93 | glaser* |
| S94 | S37 OR S38 OR S39 OR S40 OR S41 OR S42 OR S43 OR S44 OR S45 OR S46 OR S47 OR S48 OR S49 OR S50 OR S51 OR S52 OR S53 OR S54 OR S55 OR S56 OR S57 OR S58 OR S59 OR S60 OR S61 OR S62 OR S63 OR S64 OR S65 OR S66 OR S67 OR S68 OR S69 OR S70 OR S71 OR S72 OR S73 OR S74 OR S75 OR S76 OR S77 OR S78 OR S79 OR S80 OR S81 OR S82 OR S83 OR S84 OR S85 OR S86 OR S87 OR S88 OR S89 OR S90 OR S91 OR S92 OR S93 |
| S95 | S20 AND S36 AND S94 |

## ASSIA (ProQuest)

(MAINSUBJECT.EXACT("Self destructive behavior") OR MAINSUBJECT.EXACT("Self injury") OR noft((overdos* OR (over PRE/1 dos*) OR handbang* OR (head PRE/1 bang*) OR parasuicid* OR (para PRE/1 suicid*) OR (suicid* NEAR/2 (ideation* OR attempt*)) OR ((self OR selves OR themsel* OR himsel* OR hersel*) NEAR/3 harm*) OR ((self OR selves OR themsel* OR himsel* OR hersel*) NEAR/3 mutilat*) OR ((self OR selves OR themsel* OR himsel* OR hersel*) NEAR/3 injur*) OR ((self OR selves OR themsel* OR himsel* OR hersel*) NEAR/3 poison*) OR ((self OR selves OR themsel* OR himsel* OR hersel*) NEAR/3 cut*) OR ((self OR selves OR themsel* OR himsel* OR hersel*) NEAR/3 inflict*) OR ((self OR selves OR themsel* OR himsel* OR hersel*) NEAR/3 destruct*)))) AND ((MAINSUBJECT.EXACT("Rural areas") OR MAINSUBJECT.EXACT("Rural communities") OR MAINSUBJECT.EXACT("Agriculture") OR MAINSUBJECT.EXACT("Farmers") OR MAINSUBJECT.EXACT.EXPLODE("Farms") OR MAINSUBJECT.EXACT("Farmworkers")) OR noft(rural* OR countryside OR "country side" OR nonurban* OR (non PRE/1 urban*) OR remote OR village* OR farm* OR agricultur* OR husbandry OR veterinarian OR vet OR vets OR (veterinary PRE/1 (surgeon* OR nurse* OR practitioner*)))) AND (interview* OR theme* OR "thematic analysis" OR qualitative OR "nursing research methodology" OR questionnaire OR ethnograph* OR ethnonursing OR "ethnological research" OR phenomenol* OR (grounded PRE/1 (theor* OR stud* OR research OR analys?s)) OR ((life OR women's) PRE/1 stor*) OR (emic OR etic OR hermeneutic OR heuristic OR semiotic OR (data NEAR/1 saturat*) OR (participant PRE/1 observ*)) OR ((social PRE/1 construct*) OR postmodern* OR post-modern* OR poststructural* OR post-structural* OR feminis* OR interpret*) OR ("action research" OR (co-operative PRE/1 inquir*) OR (cooperative PRE/1 inquir*)) OR (humanistic OR existential OR experiential OR paradigm*) OR (field PRE/1 (stud* OR research)) OR ("human science" OR "biographical method") OR (theoretical PRE/1 sampl*) OR (purposive PRE/1 sampl*) OR (((unstructured OR open-ended) PRE/1 account*) OR text* OR narrative*) OR ("life world" OR "theoretical saturation" OR (conversation PRE/1 analys?s)) OR ((life OR lived) PRE/1 experience*) OR (cluster PRE/1 sampl*) OR (observational PRE/1 method*) OR ("content analysis" OR "constant comparative") OR ((discourse OR discurs*) PRE/1 analys?s) OR (narrative PRE/1 analys?s) OR (heidegger* OR colaizzi* OR spiegelberg* OR (van PRE/1 manen*) OR (van PRE/1 kaam*)) OR ((merleau PRE/1 ponty*) OR husserl* OR foucault* OR (corbin* NEAR/2 strauss*) OR glaser* OR (mix* NEAR/2 method*) OR (mix* NEAR/2 design*)))

## Web of Science (Science Citation Index Expanded (SCI-EXPANDED), Social Sciences Citation Index (SSCI))

| 1 | TI=((self or themsel* or himsel* or hersel*) NEAR/2 harm*) OR AB=((self or themsel* or himsel* or hersel*) NEAR/2 harm*) OR AK=((self or themsel* or himsel* or hersel*) NEAR/2 harm*) |
| --- | --- |
| 2 | TI=((self or themsel* or himsel* or hersel*) NEAR/2 mutilat*) OR AB=((self or themsel* or himsel* or hersel*) NEAR/2 mutilat*) OR AK=((self or themsel* or himsel* or hersel*) NEAR/2 mutilat*) |
| 3 | TI=((self or themsel* or himsel* or hersel*) NEAR/2 injur*) OR AB=((self or themsel* or himsel* or hersel*) NEAR/2 injur*) OR AK=((self or themsel* or himsel* or hersel*) NEAR/2 injur*) |
| 4 | TI=((self or themsel* or himsel* or hersel*) NEAR/2 poison*) OR AB=((self or themsel* or himsel* or hersel*) NEAR/2 poison*) OR AK=((self or themsel* or himsel* or hersel*) NEAR/2 poison*) |
| 5 | TI=((self or themsel* or himsel* or hersel*) NEAR/2 cut*) OR AB=((self or themsel* or himsel* or hersel*) NEAR/2 cut*) OR AK=((self or themsel* or himsel* or hersel*) NEAR/2 cut*) |
| 6 | TI=((self or themsel* or himsel* or hersel*) NEAR/2 inflict*) OR AB=((self or themsel* or himsel* or hersel*) NEAR/2 inflict*) OR AK=((self or themsel* or himsel* or hersel*) NEAR/2 inflict*) |
| 7 | TI=((self or themsel* or himsel* or hersel*) NEAR/2 destruct*) OR AB=((self or themsel* or himsel* or hersel*) NEAR/2 destruct*) OR AK=((self or themsel* or himsel* or hersel*) NEAR/2 destruct*) |
| 8 | TI=((self or themsel* or himsel* or hersel*) NEAR/2 wound*) OR AB=((self or themsel* or himsel* or hersel*) NEAR/2 wound*) OR AK=((self or themsel* or himsel* or hersel*) NEAR/2 wound*) |
| 9 | TI=((self or themsel* or himsel* or hersel*) NEAR/2 violen*) OR AB=((self or themsel* or himsel* or hersel*) NEAR/2 violen*) OR AK=((self or themsel* or himsel* or hersel*) NEAR/2 violen*) |
| 10 | TI=(auto-mutilat* or automutilat*) OR AB=(auto-mutilat* or automutilat*) OR AK=(auto-mutilat* or automutilat*) |
| 11 | TI=(overdos* OR over-dos*) OR AB=(overdos* OR over-dos*) OR AK=(overdos* OR over-dos*) |
| 12 | TI=(headbang* OR head-bang*) OR AB=(headbang* OR head-bang*) OR AK=(headbang* OR head-bang*) |
| 13 | TI=(parasuicid* OR para-suicid*) OR AB=(parasuicid* OR para-suicid*) OR AK=(parasuicid* OR para-suicid*) |
| 14 | TI=(suicid* NEAR/2 (ideation* or attempt*)) OR AB=(suicid* NEAR/2 (ideation* or attempt*)) OR AK=(suicid* NEAR/2 (ideation* or attempt*)) |
| 15 | #14 OR #13 OR #12 OR #11 OR #10 OR #9 OR #8 OR #7 OR #6 OR #5 OR #4 OR #3 OR #2 OR #1 |
| 16 | TI=rural* OR AB=rural* OR AK=rural* |
| 17 | TI=(countryside OR country-side) OR AB=(countryside OR country-side) OR AK=(countryside OR country-side) |
| 18 | TI=(nonurban* OR non-urban*) OR AB=(nonurban* OR non-urban*) OR AK=(nonurban* OR non-urban*) |
| 19 | TI=(remote OR village*) OR AB=(remote OR village*) OR AK=(remote OR village*) |
| 20 | TI=(agricultur* OR husbandary OR farm*) OR AB=(agricultur* OR husbandary OR farm*) OR AK=(agricultur* OR husbandary OR farm*) |
| 21 | TI=(veterinarian or vet or vets or (veterinary NEAR/0 (surgeon* or nurse* or practitioner*))) OR AB=(veterinarian or vet or vets or (veterinary NEAR/0 (surgeon* or nurse* or practitioner*))) OR AK=(veterinarian or vet or vets or (veterinary NEAR/0 (surgeon* or nurse* or practitioner*))) |
| 22 | #16 OR #17 OR #18 OR #19 OR #20 OR #21 |
| 23 | TI=(interview* OR theme* OR "thematic analysis") OR AB=(interview* OR theme* OR "thematic analysis") OR AK=(interview* OR theme* OR "thematic analysis") |
| 24 | TI=(qualitative OR phenomenol*) OR AB=(qualitative OR phenomenol*) OR AK=(qualitative OR phenomenol*) |
| 25 | TI="nursing research methodology" OR AB="nursing research methodology" OR AK="nursing research methodology" |
| 26 | TI=questionnaire* OR AB=questionnaire* OR AK=questionnaire* |
| 27 | TI=(ethnonursing OR ethnograph* OR "ethnological research") OR AB=(ethnonursing OR ethnograph* OR "ethnological research") OR AK=(ethnonursing OR ethnograph* OR "ethnological research") |
| 28 | TI=("grounded theor*" OR "grounded stud*" OR "grounded research" OR "grounded analysis" OR "grounded analyses") OR AB=("grounded theor*" OR "grounded stud*" OR "grounded research" OR "grounded analysis" OR "grounded analyses") OR AK=("grounded theor*" OR "grounded stud*" OR "grounded research" OR "grounded analysis" OR "grounded analyses") |
| 29 | TI=(mix* NEAR/2 (method* or design*)) OR AB=(mix* NEAR/2 (method* or design*)) OR AK=(mix* NEAR/2 (method* or design*)) |
| 30 | TI=("life stor*" OR "women's stor*") OR AB=("life stor*" OR "women's stor*") OR AK=("life stor*" OR "women's stor*") |
| 31 | TI=(emic OR etic OR hermeneutic OR heuristic OR semiotic OR (data NEAR/1 saturat*) OR "participant observ*") OR AB=(emic OR etic OR hermeneutic OR heuristic OR semiotic OR (data NEAR/1 saturat*) OR "participant observ*") OR AK=(emic OR etic OR hermeneutic OR heuristic OR semiotic OR (data NEAR/1 saturat*) OR "participant observ*") |
| 32 | TI=("social construct*" OR postmodern* OR post-modern* OR post-structural* OR poststructural* OR feminis* OR interpret*) OR AB=("social construct*" OR postmodern* OR post-modern* OR post-structural* OR poststructural* OR feminis* OR interpret*) OR AK=("social construct*" OR postmodern* OR post-modern* OR post-structural* OR poststructural* OR feminis* OR interpret*) |
| 33 | TI=("action research" OR "cooperative inquir*" OR (co-operative NEAR/1 inquir*)) OR AB=("action research" OR "cooperative inquir*" OR (co-operative NEAR/1 inquir*)) OR AK=("action research" OR "cooperative inquir*" OR (co-operative NEAR/1 inquir*)) |
| 34 | TI=(humanistic OR existential OR experiential OR paradigm*) OR AB=(humanistic OR existential OR experiential OR paradigm*) OR AK=(humanistic OR existential OR experiential OR paradigm*) |
| 35 | TI=("field stud*" OR "field research" OR "human science") OR AB=("field stud*" OR "field research" OR "human science") OR AK=("field stud*" OR "field research" OR "human science") |
| 36 | TI=("theoretical sampl*" OR "purposive sampl*" OR "biographical method*") OR AB=("theoretical sampl*" OR "purposive sampl*" OR "biographical method*") OR AK=("theoretical sampl*" OR "purposive sampl*" OR "biographical method*") |
| 37 | TI=("open-ended account*" OR "unstructured account" OR narrative* OR text*) OR AB=("open-ended account*" OR "unstructured account" OR narrative* OR text*) OR AK=("open-ended account*" OR "unstructured account" OR narrative* OR text*) |
| 38 | TI=("life world" OR "conversation analysis" OR "conversation analyses" OR "theoretical saturation") OR AB=("life world" OR "conversation analysis" OR "conversation analyses" OR "theoretical saturation") OR AK=("life world" OR "conversation analysis" OR "conversation analyses" OR "theoretical saturation") |
| 39 | TI=("lived experience*" OR "life experience*") OR AB=("lived experience*" OR "life experience*") OR AK=("lived experience*" OR "life experience*") |
| 40 | TI=("observational method*" OR "cluster sampl*") OR AB=("observational method*" OR "cluster sampl*") OR AK=("observational method*" OR "cluster sampl*") |
| 41 | TI=("content analysis" OR "constant comparative") OR AB=("content analysis" OR "constant comparative") OR AK=("content analysis" OR "constant comparative") |
| 42 | TI=("discourse analysis" OR "discourse analyses" OR "discurs* analysis" OR "discurs* analyses") OR AB=("discourse analysis" OR "discourse analyses" OR "discurs* analysis" OR "discurs* analyses") OR AK=("discourse analysis" OR "discourse analyses" OR "discurs* analysis" OR "discurs* analyses") |
| 43 | TI=("narrative analysis" OR "narrative analyses") OR AB=("narrative analysis" OR "narrative analyses") OR AK=("narrative analysis" OR "narrative analyses") |
| 44 | TI=(heidegger* OR colaizzi* OR spiegelberg* OR husserl* OR foucault* OR glaser*) OR AB=(heidegger* OR colaizzi* OR spiegelberg* OR husserl* OR foucault* OR glaser*) OR AK=(heidegger* OR colaizzi* OR spiegelberg* OR husserl* OR foucault* OR glaser*) |
| 45 | TI="van manen*" OR AB="van manen*" OR AK="van manen*" |
| 46 | TI="van kaam*" OR AB="van kaam*" OR AK="van kaam*" |
| 47 | TI="merleau ponty*" OR AB="merleau ponty*" OR AK="merleau ponty*" |
| 48 | TI=(corbin* NEAR/2 strauss*) OR AB=(corbin* NEAR/2 strauss*) OR AK=(corbin* NEAR/2 strauss*) |
| 49 | #48 OR #47 OR #46 OR #45 OR #44 OR #43 OR #42 OR #41 OR #40 OR #39 OR #38 OR #37 OR #36 OR #35 OR #34 OR #33 OR #32 OR #31 OR #30 OR #29 OR #28 OR #27 OR #26 OR #25 OR #23 OR #24 |
| 50 | #49 AND #15 AND #22 |
