# Supplementary material for: Experiences and Perceptions of Self‐Harm in Rural‐Dwelling Adults: A Rapid Review of Qualitative Evidence
Source: Health Expect. 2025 Apr 25;28(3):e70268. doi: 10.1111/hex.70268 (PMC12022502; doi:10.1111/hex.70268)
Supplement: Supplementary file 2 — FILE 002_Experiences and perceptions of self‐harm in rural‐dwelling adults _A rapid review of qualitative evidence w_Supplimentary tables. [file HEX-28-e70268-s002.docx]

| **Table 1 – Full inclusion and exclusion criteria** | | |
| --- | --- | --- |
|  | **Inclusion** | **Exclusion** |
| **Study design** | - Qualitative study of any design - Mixed methods with a qualitative component. | - Quantitative - Mixed methods without qualitative component - Editorials, letters, book reviews - Scoping reviews - Conference abstracts |
| **Participants and conditions of interest** | - Persons aged 18 years and over. - International. - Acts of self-harm (people who have experienced self-harm, people who have cared for people with experience of personal self-harm). - Attempted suicide. - Drug use with the intention of harm. - Overdose with the intention to harm. | - Persons aged 17 years and younger. - Suicidal ideation with no act of self-harm - Assisted suicide. - Habitual drug use. - Accidental overdose with no intention to harm. |
| **Interventions** | Any |  |
| **Outcomes** | - Experiences of self-harm in rural setting. - Social factors influencing access to health care. - Environmental factors influencing access to health care. - Other factors influencing access to health care. |  |
| **Setting** | Rural settings* in any country | Urban settings |
| **Other** | English Language |  |

| **Table 2. MMAT** |  | |  | | | | |  |  | | | | |  |
| --- | --- | --- | --- | --- | --- | --- | --- | --- | --- | --- | --- | --- | --- | --- |
|  | **Screening** | | **Qualitative Studies** | | | | |  | **Mixed Methods** | | | | |  |
| First Author & Year | S1. | S2. | 1.1 | 1.2 | 1.3 | 1.4 | 1.5 | 5.1 | | 5.2 | 5.3 | 5.4 | 5.5 | **Overall score** |
| Ataley Alem, 1999 | Yes | Yes | 1 | 1 | 1 | 1 | 0 | N/A | | N/A | N/A | N/A | N/A | 4/5 80%  Moderate- Strong |
| Veronica Pearson, 2002 | Yes | Yes | 1 | 1 | 1 | 1 | 0 | 0 | | 1 | 0 | 0 | 0 | 5/10 50%  Moderate - Weak |
| Janine Slaven, 2002 | Yes | Yes | 1 | 1 | 1 | 1 | 1 | N/A | | N/A | N/A | N/A | N/A | 5/5 100%  Strong |
| Lalith Senarathna, 2008 | Yes | Yes | 1 | 1 | 1 | 1 | 1 | N/A | | N/A | N/A | N/A | N/A | 5/5 100%  Strong |
| Zelda Holtman, 2011 | Yes | Yes | 1 | 1 | 1 | 1 | 1 | N/A | | N/A | N/A | N/A | N/A | 5/5 100%  Strong |
| Lalith Senarathna, 2013 | Yes | Yes | 1 | 1 | 1 | 1 | 1 | N/A | | N/A | N/A | N/A | N/A | 5/5 100%  Strong |
| Genevieve Creighton, 2017 | Yes | Yes | 1 | 1 | 1 | 1 | 1 | N/A | | N/A | N/A | N/A | N/A | 5/5 100%  Strong |
| Anton N. Isaacs, 2017 | Yes | Yes | 1 | 1 | 1 | 1 | 1 | N/A | | N/A | N/A | N/A | N/A | 5/5 100%  Strong |
| Meg Perceval, 2018 | Yes | Yes | 1 | 1 | 1 | 1 | 1 | N/A | | N/A | N/A | N/A | N/A | 5/5 100%  Strong |
| Meg Perceval, 2019 | Yes | Yes | 1 | 1 | 1 | 1 | 1 | N/A | | N/A | N/A | N/A | N/A | 5/5 100%  Strong |
| Kimberly A. Link, 2020 | Yes | Yes | 1 | 1 | 1 | 1 | 1 | N/A | | N/A | N/A | N/A | N/A | 5/5 100%  Strong |
| Daniel R. George,2021 | Yes | Yes | 1 | 1 | 1 | 1 | 1 | N/A | | N/A | N/A | N/A | N/A | 5/5 100%  Strong |
| Scott J. Fitzpatrick, 2021 | Yes | Yes | 1 | 1 | 1 | 1 | 1 | 1 | | 1 | 1 | 1 | 1 | 10/10 100%  Strong |
| Angie Waliski, 2023 | Yes | Yes | 1 | 1 | 1 | 1 | 1 | N/A | | N/A | N/A | N/A | N/A | 5/5 100%  Strong |

| **Table 3. List of included articles** | |  |  |  |  |
| --- | --- | --- | --- | --- | --- |
| First author & Year | Study Type & Methodology | Participants | Setting | Reason for inclusion | Number |
| Ataley Alem, 1999 | Qualitative interviews | 88 | Rural Ethiopia | Suicidal behaviour (including self-harm) | [1] |
| Veronica Pearson, 2002 | Mixed Methods:  Descriptive statistics and interviews | 147 patients  138 family | Rural China | Attempted suicide. | [2] |
| Janine Slaven, 2002 | Qualitative, semi-structured interviews | 25 | Rural western Australia | Deliberate self-harm | [3] |
| Lalith Senarathna, 2008 | Qualitative semi-structured interviews | 15 | Rural Sri Lanka | Self-harm (self-poisoning) | [4] |
| Zelda Holtman, 2011 | Qualitative, semi-structured interviews | 10 | ‘Small town’ in South Africa | Suicide (self-harm discussed) | [5] |
| Lalith Senarathna, 2013 | Qualitative, focus groups | 17 doctors, 35 nurses, and 58 minor staff members | Rural Sri Lanka | Self-harm (self-poisoning) | [6] |
| Genevieve Creighton, 2017 | Qualitative, semi-structured interviews and photovoice. | 15 | Rural Canada | Perceptions of self-harm discussed | [7] |
| Anton N. Isaacs, 2017 | Qualitative interviews. | 27 | Rural Australia | Suicide (self-harm discussed) | [8] |
| Meg Perceval, 2018 | Qualitative, focus groups | 66 | Rural Australia | Suicide attempts | [9] |
| Meg Perceval, 2019 | Qualitative, focus groups | 27 | Rural Australia. | Mention of fatal self-harm | [10] |
| Kimberly A. Link, 2020 | Qualitative interviews | 3 | Rural southeast USA | Historical suicide attempts. | [11] |
| Daniel R. George,2021 | Qualitative, four focus groups | 60 | Rural and urban USA | Suicide and self-harm (diseases of despair) | [12] |
| Scott J. Fitzpatrick, 2021 | Mixed methods:  Descriptive statistics and case studies. | 792 case reports for quant.  30 cases for qual. | Rural Australia | Historical suicidal thoughts | [13] |
| Angie Waliski, 2023 | Qualitative semi-structured interviews | 10 | Rural Arkansas, USA | Suicide attempts | [14] |

***** No current universal rural definition, rural setting is accepted on the basis that included papers label their population as rural.

| **Table 4 – Theme development** | | |
| --- | --- | --- |
| Author and year | In article themes | Overlapping themes |
| Ataley Alem, 1999 | Reasons for suicide/ attempted suicide.  Community and cultural perceptions of suicide/ attempted suicide. | Reasons for self-harm,  Community perceptions of self-harm. |
| Veronica Pearson, 2002 | Reasons for self-poisoning. | Reasons for self-harm  Lack of support and resources. |
| Janine Slaven, 2002 | Management of patients presenting to hospital with DSH.  Barriers to effective management according to nursing staff.  Barriers to effective management according to GPs.  Barriers to effective management according to MHPs | HCP perceptions of self-harm  Lack of support and resources |
| Lalith Senarathna, 2008 | Conceptualising self-poisoning as a health problem.  Staff and community relations.  Resources and locations:  Experience improves focus and confidence in treating and or transferring self-poisoned patients. | Lack of support and resources.  HCP perceptions of self-harm. |
| Zelda Holtman, 2011 | Proposed predisposing factors  Factors perceived to precipitate a suicide attempt.  Methods of self-harm  Support provided after the suicide attempt | Reasons for self-harm  Lack of support and resources. |
| Scott J. Fitzpatrick, 2021 | Mental health and suicide  Physical health and suicide.  Socioeconomic and psychosocial circumstances and suicide.  Care practices. | Reasons for self-harm  HCP perceptions of self-harm.  Lack of support and resources. |
| Lalith Senarathna, 2013 | Interactions between the community and the hospital  Healthcare teamwork in rural peripheral hospitals  Doctors: Decision makers with limited options and resources.  Doctors and nurses: shared decision making.  Minor staff categories: unofficial communication mediators.  Staff perceptions of peripheral hospitals. | Lack of support and resources.  Community perceptions of self-harm.  HCP perceptions of self-harm |
| Genevieve Creighton, 2017 | Hiding depression and its cause  Manly self-medicating  Mobilizing prevention | Reasons for self-harm  Community perceptions of self-harm |
| Anton N. Isaacs, 2017 | Difficulty in talking about one’s problems.  Reasons for not talking to family and peers.  Lack of access to suitable formal supports.  Consequences of not talking about ones problems. | Lack of support and resources.  Community perceptions of self-harm  Reasons for self-harm |
| Meg Perceval, 2018 | Reasons for suicide:  Changing rural communities  Community attitudes and sigma  Relationship issues | Reasons for self-harm  Community perceptions of self-harm. |
| Meg Perceval, 2019 | Reasons for suicide:  Extreme climatic events  Isolation  Service availability  Access to and frequent use of firearms.  Death and suffering of animals  Government and legislation  Technology  Property values. | Reasons for self-harm  Lack of support and resources. |
| Kimberly A. Link, 2020 | Family members expressed guilt about not being able to prevent suicide.  Family members experienced symptoms of psychological distress.  Family members felt attached to the land, immediate coping strategies post suicide.  Long term coping skills after suicide.  Lack of community support. | Lack of support and resources.  Reasons for self-harm. |
| Daniel R. George,2021 | Reasons for mental health decline (suicide, self-harm inclusive).  Intervention strategies. | Reasons for self-harm |
| Scott J. Fitzpatrick, 2021 | Mental health and suicide  Physical health and suicide.  Socioeconomic and psychosocial circumstances and suicide.  Care practices. | Reasons for self-harm  HCP perceptions of self-harm.  Lack of support and resources. |
| Angie Waliski, 2023 | Perceptions of suicide attempt method.  Setting, intervention, and individual characteristics: current practices and procedures.  Implementation process: Recommendations to improve ED care for all suicidal patients. | Lack of support and resources.  HCP attitudes towards self-harm. |

| Table 5. Grades of Recommendation, Assessment, Development, and Evaluation Confidence in the Evidence from Qualitative Reviews summary of qualitative findings. | | | | | |
| --- | --- | --- | --- | --- | --- |
|  | Methodological limitations | Coherence | Adequacy | Relevance | CERQual confidence assessment |
| Review finding (and contributing studies) | Concerns |  |  |  |  |
| Theme 1 – Experiences of self-harm. |  |  |  |  |  |
| Subtheme 1: Reasons for self-harm: Marital conflict, loneliness, and isolation.  Personal factors that may have contributed to self-harm were discussed in the majority of papers, examples of this are marital stress, loneliness, isolation etc.  1, 2, 3, 4, 5, 6, 8, 11, 12, 14 | **Minimal or no concerns:**  All papers used effective methodology.  Some papers used small sample size (2 people). | **Minor coherence concerns:**  Data is consistent within and across studies. | **Minor adequacy concerns:**  Low number of participants in some studies, but still provides rich data in combination with other studies. | **Very minor relevancy concerns:**  One study discussed both urban and rural thoughts as one [1] but across studies it provides a coherent picture. | **High confidence:**  Rated high as these 11 studies provide a clear picture of the personal reason why people may self-harm in rural communities. |
| Subtheme 1: Reasons for self-harm: Experiences of illness.  Medical reasons such as ill health, alcoholism, psychological issues, and drugs were also discussed in the papers.  1, 2, 3, 5, 8, 10, 12, 14. | **Minimal or no concerns:**  All papers used effective methodology.  Some papers used small sample size (2 people). | **Minor coherence concerns:**  Data is consistent within and across studies**.** | **Minor adequacy concerns:**  Low number of participants in some studies, but still provides rich data in combination with other studies. | **Minor relevancy concerns:**  Some studies imply medical reasons (mention drugs or mental health). Rather than state outright this is a reason. | **High confidence:**  Rated high as these 9 studies provided clear picture that medical reason maybe a predictor for self-harm, although some of these reasons may be implied**.** |
| Subtheme 1: Reasons for self-harm: rural associated occupations and area level decline.  Area level factors such as access to means, loneliness, isolation, droughts, government funding blocks were discussed in the studies.  2, 5, 7, 8, 11, 12 | **Minimal or no concerns:**  All papers used effective methodology.  Some papers used small sample size (2 people). | **Minor coherence concerns:**  Data is consistent within and across studies**.** | **Minor adequacy concerns:**  Low number of participants in some studies, but still provides rich data in combination with other studies. | **Very minor relevancy concerns:**  One study discussed both urban and rural thoughts as one [1] but across studies it provides a coherent picture. | **High confidence:**  Rated high as these 6 studies provide a clear picture of the area level reasons why people may self-harm in rural communities. |
| Subtheme 2: Perceptions of self-harm: Cultural stigma.  Cultural stigma of smaller rural communities was discussed amongst the papers, this linked to religious practices – as well as cultural stigma, fear of weakness and fear of judgement.  2, 3, 4, 5, 11, 14 | **Minimal or no concerns:**  All papers used effective methodology.  Some papers used small sample size (2 people). | **Minor coherence concerns:**  Data is consistent within and across studies**.** | **Minor adequacy concerns:**  Low number of participants in some studies, but still provides rich data in combination with other studies. | **Minor relevancy concerns:**  Some studies mention culture explicitly in terms of cultural background, others discuss culture as in community cultures. | **High confidence:**  Rated high as these 7 studies highlight the attitudes and stigmas across cultures. |
| Subtheme 2: Community-held perceptions of self-harm: Gender differences (stoic men).  The expectation of men such as the added pressures of being ‘stoic’ was discussed in the papers, including the weakness associated with poor mental health, suicide, and self-harm.  1, 4, 5, 14 | **Minimal or no concerns:**  All papers used effective methodology. | **Minor coherence concerns:**  Data is consistent within and across studies**.** | **Very minor adequacy concerns:**  Rich data has been sourced across studies. | **Minor relevancy concerns:**  1 study [12] mentions about woman being different rather than explicitly mentioning men, but end point the same. | **High confidence:**  Rated high as these 6 studies effectively discuss gender differences, in particular stoic male attitudes. |
| Theme 2: Accessing to care. |  |  |  |  |  |
| Subtheme 1: Healthcare provider perceptions of self-harm: judgemental attitudes.  Judgmental attitudes of healthcare providers were discussed, including things not being their responsibility and not believing patients that present with problems.  1, 7, 9, 10 | **Minimal or no concerns:**  All papers used effective methodology, with focus on healthcare providers as participants. | **Minor coherence concerns:**  Data is consistent within and across studies**.** | **Minor adequacy concerns:**  Studies provide rich data sourced across all studies. | **Moderate relevancy concerns:**  In some studies, the judgmental attitudes are implied rather than being stated outright. [6&9] | **High confidence:**  Rated high as these 4 studies effectively highlight judgmental attitudes of healthcare staff, in either a direct or implied way. |
| Subtheme 1: Healthcare provider perceptions of self-harm: Lack of awareness.  The lack of awareness and options for professional progression were discussed by health care providers in the studies.  2, 6, 7, 9, 10, 13, 14 | **Minimal or no concerns:**  All papers used effective methodology, with focus on healthcare providers as participants. | **Minor coherence concerns:**  Data is consistent within and across studies**.** | **Minor adequacy concerns:**  Studies provide rich data sourced across all studies. | **Moderate relevancy concerns:**  In most studies lack of awareness was implied rather than stated outright. | **High/Moderate confidence:**  Rated high/ moderate as these 7 studies highlight that there is a lack of awareness for mental health in healthcare workers. |
| Subtheme 2: Lack of resources: Lack of informal support.  The lack of informal support was noted in the papers, with things such as confidence is speaking out about mental health, not feeling like they can talk to friends/ family and community about struggles. Or mental health in general.  1, 2, 3, 4, 5, 7, 8, 11, 12, 14, | **Minimal or no concerns:**  All papers used effective methodology. | **Minor coherence concerns:**  Data is consistent within and across studies**.** | **Minor adequacy concerns:**  Studies provide rich data sourced across all studies. | **Moderate relevancy concerns:**  One study [4] discusses thought on talking about suicide rather than discussing directly, mentions lack of informal support. Overall, a coherent picture. | **High/Moderate confidence:**  Rated high/ moderate as these 11 studies highlight a lack of informal support. As one study discussed this through a Third party [4]. |
| Subtheme 2: Lack of resources: Lack of formal support.  The lack of mental health resources, training and opportunity for support was noted across the studies.  2, 4, 5, 6, 7, 8, 9, 10, 11, 12, 13, 14 | **Minimal or no concerns:**  All papers used effective methodology. | **Minor coherence concerns:**  Data is consistent within and across studies**.** | **Minor adequacy concerns:**  Studies provide rich data sourced across all studies. | **Moderate relevancy concerns:**  One study [4] discusses thought on talking about suicide rather than discussing directly, mentions lack of formal support. Overall, a coherent picture. | **High/Moderate confidence:**  Rated high/ moderate as these 13 studies highlight a lack of formal support. As one study discussed this through a Third party [4]. |
| Subtheme 2: Lack of resources: Lack of infrastructure.  The lack of infrastructure such as telecommunications, public transport and others were also discussed.  2, 4, 5, 7, 8, 10, 12, | **Minimal or no concerns:**  All papers used effective methodology. | **Minor coherence concerns:**  Data is consistent within and across studies**.** | **Minor adequacy concerns:**  Studies provide rich data sourced across all studies. | **Moderate relevancy concerns:**  One study [4] discusses thought on talking about suicide rather than discussing directly, Mentions lack of resources. Overall, a coherent picture. | **High/Moderate confidence:**  Rated high/ moderate as these 8 studies highlight a lack of resources. As one study discussed this through a Third party [4]. |
